# Non-Mendelian Dominant Maternal Effects Caused by CRISPR/Cas9 Transgenic Components in *Drosophila melanogaster*

**DOI:** 10.1534/g3.116.034884

**Published:** 2016-09-16

**Authors:** Chun-Chieh Lin, Christopher J. Potter

**Affiliations:** The Solomon H. Snyder Department of Neuroscience, Johns Hopkins University School of Medicine, Baltimore, Maryland 21205

**Keywords:** gene drive, maternal effect, HACK, mutagenic chain reaction, MCR

## Abstract

The CRISPR/Cas9 system has revolutionized genomic editing. The Cas9 endonuclease targets DNA via an experimentally determined guide RNA (gRNA). This results in a double-strand break at the target site . We generated transgenic *Drosophila melanogaster* in which the CRISPR/Cas9 system was used to target a *GAL4* transgene *in vivo*. To our surprise, progeny whose genomes did not contain CRISPR/Cas9 components were still capable of mutating *GAL4* sequences. We demonstrate this effect was caused by maternal deposition of Cas9 and gRNAs into the embryo, leading to extensive *GAL4* mutations in both somatic and germline tissues. This serves as a cautionary observation on the effects of maternal contributions when conducting experiments using genomically encoded CRISPR/Cas9 components. These results also highlight a mode of artificial inheritance in which maternal contributions of DNA editing components lead to transmissible mutant defects even in animals whose genomes lack the editing components. We suggest calling this a dominant maternal effect to reflect it is caused by the gain of maternally contributed products. Models of CRISPR-mediated gene drive will need to incorporate dominant maternal effects in order to accurately predict the efficiency and dynamics of gene drive in a population.

The phenotype of a developing animal is not determined solely by the chromosomes inherited from each parent. The female gamete provides the early cytoplasmic environment crucial for the developing embryo (Bate and Arias 1993). This cytoplasm contains various organelles, RNA, and proteins as determined by the mother’s genomic and mitochondrial DNA which are essential for proper development. This maternal effect on development was highlighted in saturating mutational screens in *Drosophila* aimed at identifying genes required for normal development (Nüsslein-Volhard and Wieschaus 1980). Mutant mothers, even when crossed to a nonmutant father, produced embryos exhibiting profound defects, such as in the establishment of the body axes during early embryonic development (Perrimon *et al.* 1984; Schüpbach and Wieschaus 1986; Schüpbach and Wieschaus 1991). Products of these maternal effect genes (RNA and protein) are deposited into the egg where they play direct roles in the development of a fertilized embryo. Maternal effect inheritance is defined that all progeny from a mutant mother will show a mutant phenotype, even if the developing embryo contains a functional gene inherited from the father (Nüsslein-Volhard and Wieschaus 1980).

Gene editing techniques have been revolutionized in recent years by the introduction of the clustered regularly interspaced short palindromic repeat (CRISPR)/Cas9 system (Jinek *et al.* 2012; Bassett *et al.* 2013; Cong *et al.* 2013; Gratz *et al.* 2013; Mali *et al.* 2013; Doudna and Sontheimer 2014). This system, originally used in bacteria as a native defense mechanism against viral infection, utilizes the Cas9 class of proteins to generate a double-strand DNA break. The binding of Cas9 to DNA is directed by a 20 nucleotide guide RNA (gRNA), whose only restriction is the presence of a protospacer adjacent motif (PAM) sequence (Jinek *et al.* 2012; Mali *et al.* 2013). Given the adaptability of this system to target essentially any DNA sequence via an experimentally introduced small RNA, it has been rapidly adapted and validated as an effective genome editing system in many organisms, such as bacteria, worms, insects, and mammals (Doudna and Charpentier 2014). To date, most CRISPR/Cas9-mediated gene editing is achieved by microinjection of a DNA vector (vectors expressing Cas9 endonuclease and gRNA), RNA (gRNAs), or protein (Cas9 endonuclease) (Mashiko *et al.* 2013; Yang *et al.* 2013; Gratz *et al.* 2015). To increase the levels of gRNAs and Cas9 protein expressed in a target cell, which could also increase targeting efficiencies, Cas9- and gRNA-expressing cells lines and organisms have been generated. This has recently led to fully CRISPR/Cas9 genetically encoded systems to create insertion/deletion (indel) mutations or knockins (Kondo and Ueda 2013; Port *et al.* 2014; Gantz and Bier 2015; Lin and Potter 2016). Since the two major components (Cas9 and gRNAs) in the CRISPR/Cas9 system might be preloaded during oogenesis, the system could potentially generate inheritance patterns analogous to a maternal effect (Port *et al.* 2014; Gantz *et al.* 2015).

Here, we demonstrate an example of non-Mendelian maternal inheritance driven in a genetically encoded CRISPR/Cas9 organism. We found that maternally deposited CRISPR/Cas9 components (Cas9 and gRNAs) led to targeted mutations even in animals genomically lacking these genes. Furthermore, the targeted mutations were effectively transmitted to the majority of progeny regardless of the genotype of the progeny. This suggests that genetically encoded CRISPR/Cas9 techniques, such as mutagenic chain reaction (MCR), CRISPR-mediated gene drive, or Homology Assisted CRISPR Knockin (HACK), can affect the genome of progeny lacking DNA editing components. It is warranted to consider these dominant maternal effects when analyzing animals generated by these methods or models predicting their outcomes.

## Materials and Methods

### Drosophila genetics

The fly stocks used in the study are *5xUAS-GFPnls* (BS#4775), *5xQUAS-nucLacZ* (Potter *et al.* 2010) (BS#30006), *10xQUAS-6xGFP* (Shearin *et al.* 2014) (BS#52264), *5xUAS-mtdt-3HA* (Potter *et al.* 2010), *Act5C-Cas9* (Port *et al.* 2014) (BS#54590), *GMR57C10-GAL4* (BS#39171), *GMR57C10-QF2^G4H^* (BS#66479), and *attP2-QF2^G4H^* (negative orientation) (Lin and Potter 2016) (BS#66504) .

### Nervous system dissection and immunohistochemistry

Dissection of the adult *Drosophila* nervous system was performed as described previously (Lin *et al.* 2015). Brains of 4–6 d-old flies were dissected in 1× PBS solution and then fixed in 4% PFA solution (dissolved in PBS with 0.3% Triton X-100) for 15 min. Fixed tissues were washed briefly three times with PBT (PBS with 0.3% Triton X-100) before incubating in PBT for 20 min for two times. Next, 5% NGS (normal goat serum dissolved in PBT) was used as blocking solution for 30-min washing. The tissues were then placed in primary antibody mixes for 1–2 d, followed by PBT washes for 20 min for two times. The tissues were placed in secondary antibody mixes for 1 d in the dark. The following day the tissues were washed in PBT for 20 min and placed in mounting solution (Slow Fade Gold) for at least 1 hr at room temperature before imaging. All previous steps were performed at room temperature.

Immunostaining was used to enhance fluorescence signals. For GFP, the primary antibodies were rabbit anti-GFP (1:250, #A11122; Life Technologies) and chicken anti-GFP (1:1000, #GFP1020; Aves Labs Inc.). Secondary antibodies were Alexa 488 anti-rabbit (1:200, #A11034; Invitrogen) and Alexa 488 anti-chicken (1:200, #A11039; Invitrogen). For the nuclear *Lac*Z staining, the primary antibody was preabsorbed rabbit anti-*Lac*Z (1:50, #559761; MP Biochemicals). The secondary antibody was Alexa 649 anti-rabbit (1:200, #DI-1649; Vector Laboratories). To avoid the conflicting red colors from *UAS-mtdt-3HA* and *3xP3-RFP* (transgene marker), the *mtdt-3HA* was counterstained with rat anti-HA (1:250, #11867423001; Roche) as the primary antibody and Alexa 633 anti-rat (1:200, #A21094; Invitrogen) as the secondary antibody. In order to visualize the structure of the nervous system, mouse anti-nc82 (1:25, Developmental Studies Hybridoma Bank (DSHB) was used as the primary antibody and Cy3 anti-mouse (1:200, #115-165-166; Jackson ImmunoResearch) as the secondary antibody.

### Drosophila embryo immunohistochemistry

To directly detect Cas9 proteins in *Drosophila* embryos, 4-d-old parental flies of specific genotypes (see Supplemental Material, Figure S2 for details) were transferred to grape agar plates with fresh yeast paste and allowed to lay eggs for 2 hr at room temperature. The embryos were collected in a wash solution [0.7% NaCl^+^ 0.3% Triton X-100 in double-distilled H_2_O (ddH_2_O)] followed by two rinses in wash solution and then ddH_2_O. Embryos were dechorionated in a 50% commercial bleach solution (Elite Professional Bleach) for 2–3 min. Bleach was removed and wash solution added until the embryos began to sink at which point they were rinsed twice with ddH_2_O. The embryos were fixed in a 1:1 ratio of 4% PFA solution (dissolved in PBS) and heptane (, #246654; Sigma-Aldrich) and shaken for 30 sec for better penetration, and followed by a 25-min fixation on a rotator. The fixative was replaced (lower phase) with methanol and the embryos devitellinized by shaking for 15–20 sec. The devitellinized embryos sank to the bottom. The methanol and heptane and floating nondevitellinized embryos were aspirated. The embryos were rinsed three times with methanol and proceed to immunostaining steps, as described above. Mouse anti-Cas9 antibody (1:500, #ab191468; Abcam) was used as the primary antibody and Cy3 anti-mouse (1:200, #115-165-166; Jackson ImmunoResearch) as the secondary antibody.

### Confocal imaging and image processing

Confocal images were taken on a Zeiss LSM 710 confocal microscope equipped with 10× (Zeiss, Fluar 10×/0.5) and 40× (Zeiss, Plan-Apochromat 40×/1.3 Oil DIC) objectives. Zen 2012 software was used for image acquisition. For illustration purposes, Z-stack images were collapsed onto a single image by ImageJ using maximum-intensity projection and pseudocolored into different acquisition channels using an RGB plug-in.

### Cell counting

Image analysis was performed using ImageJ 1.5 (National Institutes of Health). A total of 3–4 single sections of 5-µm thick brain confocal images were collapsed into a single image, followed by color inversion (edit/invert) and threshold adjustment (image/adjust/threshold; setting to black & white). The processed image was then used for automated counting (analyze/analyze particle). The same procedures were performed for successive 3–4 sections until the whole tissue had been analyzed. Counts were summated to provide an estimated final count of labeled cells for the sample.

### Data availability

All *Drosophila* lines are available at the Bloomington Drosophila Stock Center or upon request. 

## Results and Discussion

**Figure 1:**
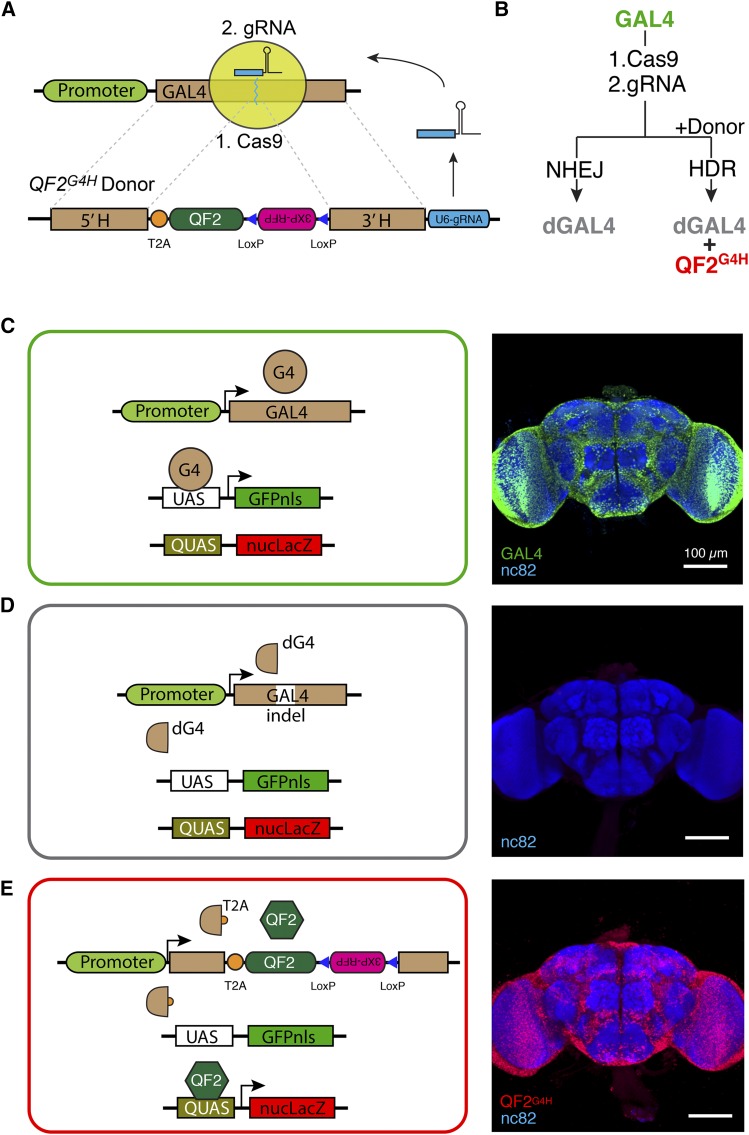
CRISPR/Cas9 system for targeting *GAL4*. (A) The CRISPR/Cas9 system consists of two components: (1) the Cas9 endonuclease and (2) the gRNA to direct Cas9 to target DNA. The *GAL4* gene is the target for the gRNA. An integrated *QF2^G4H^* donor allows *T2A-QF2* to be inserted into *GAL4* genes targeted by the CRISPR/Cas9 system. (B) Schematic of experimental outcomes. The targeting of *GAL4* by Cas9/gRNAs leads to NHEJ and a disrupted *GAL4* (*dGAL4*). In the presence of the *QF2^G4H^* donor, HDR may occur leading to QF2 expression and *dGAL4*. (C–E) Schematic and brain immunohistochemistry examples of experimental genotypes. (C) Functional GAL4 leads to *UAS-GFPnls* reporter expression in all neurons (*GMR57C10-GAL4 > UAS-GFPnls*, *QUAS-nucLacZ*). (D) Control experiment of no active *GAL4* or *QF2* transgenes (*UAS-GFPnls*, *QUAS-nucLacZ*). (E) Insertion of *T2A-QF2* into *GAL4* leads to *QUAS-nucLacZ* but not *UAS-GFPnls* expression (*GMR57C10-QF2^G4H^ > UAS-GFPnls*, *QUAS-nucLacZ*). *UAS-GFPnls* and *QUAS-nucLacZ* reporters were used as proxies for CRISPR/Cas9-induced genetic targeting of *GAL4*. Scale bars for brain images, 100 µm.

An unusual maternal inheritance was identified in experiments using the recently developed HACK technique (Lin and Potter 2016), in which gRNAs are supplied with a donor transgene compatible for homology directed repair (HDR) with a genomically targeted location (Figure 1) . In the GAL4 > QF2 HACK variant (*QF2^G4H^* donor), the method expresses gRNAs that target *GAL4* sequences for double-strand breaks, along with a donor transgene containing *GAL4* homology arms flanking a *T2A-QF2* cassette (Figure 1A). In the presence of a ubiquitous Cas9 transgene (*Actin5C-Cas9*), *GAL4* transgenes in the germline were “HACKed” to produce progeny expressing functional T2A-QF2 instead of functional GAL4. Upon a double-strand break triggered by the HACK system, *GAL4* sequence could be disrupted by non-homologous end joining (NHEJ) or converted into *QF2^G4H^* by HDR (Figure 1B). Interestingly, since CRISPR/Cas9 components were expressed ubiquitously (*Actin5C* driving Cas9 and the RNA polymerase III promoter *U6* driving gRNAs), genomic HACKing could take place in somatic cells as well (Figure 1C). To visualize the status of *GAL4* in individual cells, a dual reporter system was incorporated into all HACK genetic crosses (Figure S1). By using *UAS-GFPnls* (green fluorescent reporter) and *QUAS-nucLacZ* (stained with far-red fluorescent dye and pseudocolored red) to monitor the activity of GAL4 and QF2, respectively, we could distinguish intact GAL4 (Figure 1C, green), disrupted *GAL4* (*dGAL4*, Figure 1D, no color), and QF2^G4H^ (Figure 1E, red). Therefore, the activity of GAL4 or QF2 to drive *UAS* or *QUAS* reporter expression could be used as a proxy to monitor the activity of CRISPR/Cas9 genomic editing on somatic tissues encoding GAL4.

**Figure 2:**
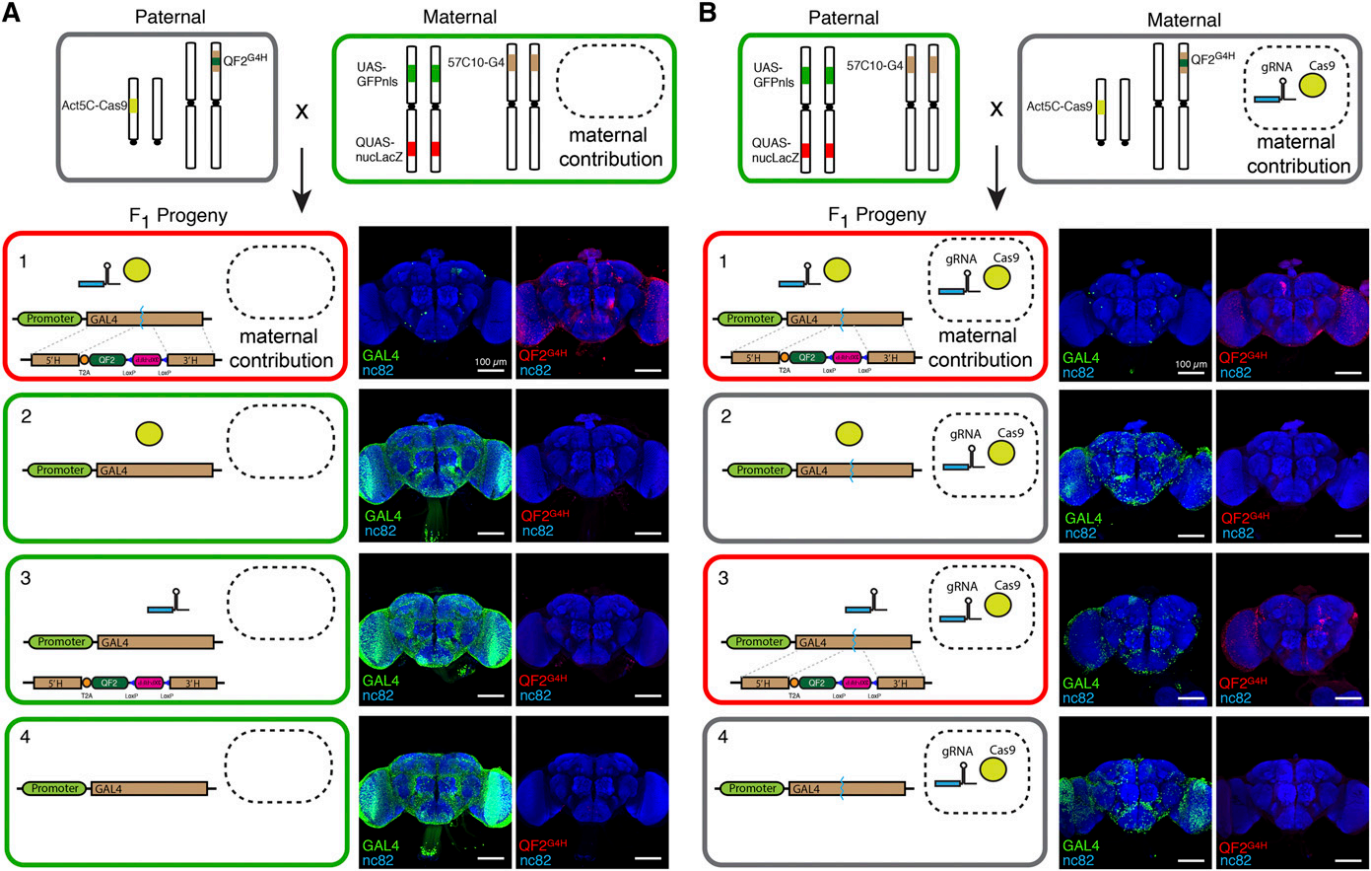
Maternal contributions of Cas9 and *GAL4*-targeting gRNAs lead to somatic mutations in *GAL4*. (A, B) Parental crosses were established to generate progeny that genetically encoded either (1) both Cas9 and GAL4-targeting gRNAs, (2) only Cas9, (3) only gRNA, or (4) neither component. Maternal contributions of Cas9 and gRNAs were experimentally controlled by the maternal genotypes. The *GMR57C10-GAL4* line expresses in all neurons, and mutation of *GAL4* was monitored by changes in *UAS-reporter* expression. Note that progeny of genotype 4 do not contain genetically encoded Cas9 or gRNAs, yet contain mutated GAL4 if these components were present in the (B) maternal parent. Genotypes: (1) *Act5C-Cas9*; *UAS-GFPnls,QUAS-nucLacZ*; *GMR57C10-GAL4/attP2-QF2^G4H^*; (2) *Act5C-Cas9*; *UAS-GFPnls,QUAS-nucLacZ*; *GMR57C10-GAL4/Tm6B*; (3) *UAS-GFPnls,QUAS-nucLacZ* ; *GMR57C10-GAL4/attP2-QF2^G4H^*; and (4) *UAS-GFPnls*, *QUAS-nucLacZ*; *GMR57C10-GAL4/Tm6B*. Scale bars for brain images, 100 µm.

To determine the effectiveness of somatic HACKing in the nervous system, we used a pan-neuronal *GAL4* line (*GMR57C10-GAL4*) as the target of Cas9 and the *attP2-QF2^G4H^* donor line (Lin and Potter 2016) (Figure 2). The adult *Drosophila* brain is estimated to contain ∼100,000 neurons (Chiang *et al.* 2011). We observed very few nuclear GFP-labeled neurons (*GAL4 > UAS-GFPnls*, 20.6 ± 2.97, *n* = 5) and ∼0.8% of neurons labeled with nuclear *Lac*Z (*QF2^G4H^ > QUAS-nucLacZ*, 800 ± 55, *n* = 5) in the adult brains. These results indicated that the genomic *GAL4* sequences of most neurons were disrupted into *dGAL4* through NHEJ, and some neurons had *GAL4* sequences HACKed into *T2A-QF2* (Figure 2, A1 and B1). Disruption of *GAL4* required the CRISPR/Cas9 components to be present in the genome (Figure 2, A2–A4).

Strikingly, this was not the case if the parental cross was simply reversed and mothers now contained *Act5C-Cas9* and *U6*-gRNAs (Figure 2B and Figure S1). We found progeny with disrupted *GAL4* transgenes even when *U6-gRNAs*, *Act5C-Cas9*, or both were absent in the offspring genome (Figure 2, B2–B4). For example, in the complete absence of genetically encoded CRISPR/Cas9 components, >90% of somatic neurons still contained *dGAL4* (GFP cell count = 810 ± 174, *n* = 2) (Figure 2B4). Since the maternal genome contains both CRISPR/Cas9 components, we reasoned that offspring somatic *GAL4* genes were targeted by maternally contributed gRNAs and Cas9 endonuclease present in the female gamete (egg). This is supported by the observation that GFP can be deposited into embryos by a maternal *Act5C-GFP* transgene (Reichhart and Ferrandon 1998). To directly verify the presence of Cas9 protein in the eggs from the *Act5C-Cas9* transgene, we performed anti-Cas9 embryo immunostaining at early developmental stages (0–2 hr after egg laying). Indeed, Cas9 protein was observed only in embryos when parental crosses contained a maternal *Act5C-Cas9* transgene, but not in those in which the *Act5C-Cas9* transgene originated from the paternal side (Figure S2).

The development of the *Drosophila* oocyte is arrested twice during meiosis: prophase I and metaphase I (Bate and Arias 1993). Once activated and ovulated, the oocyte completes meiosis, finalizing the zygotic genotype, and continues with differentiation and development. Given the short period between meiosis completion and zygote cell division, it is likely that residual gRNAs and Cas9 proteins (and mature Cas9 mRNA) remain functional and generate double-strand breaks at the target *GAL4* sequence in the dividing progenitor cells. The extent of *GAL4* disruptions from maternal contributions was enhanced by the presence of CRISPR/Cas9 transgenes in the progeny genome, which would continue to supplement gRNA and/or Cas9 endonuclease during development (Figure 2, B2 and B3). The HACK system is compatible with maternal CRISPR/Cas9 contributions such that if only *QF2^G4H^* donor (but not the Cas9 transgene) is present in the zygotic genome (*e.g.*, *attP2-QF2^G4H^*), HDR-mediated gene conversion of *GAL4* to *QF2^G4H^* can occur (Figure 2, B1 and B3).

**Figure 3:**
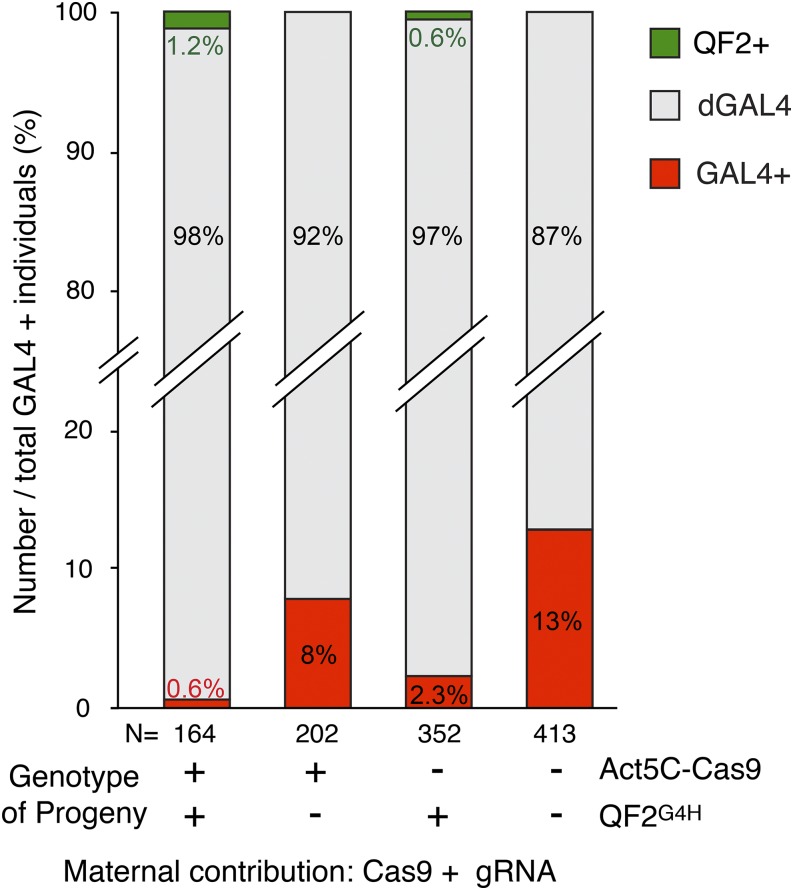
Maternal contributions of Cas9 and *GAL4*-targeting gRNAs lead to inherited mutations in *GAL4*. Male progeny of the four genotypes shown in Figure 2B were crossed to *UAS-mtdT3xHA* + *QUAS-6xGFP* reporters to determine the extent of germline *GAL4* alterations. The percentage of progeny from this cross that contained functional GAL4 (GAL4+), disrupted *GAL4* (*dGAL4*), or HDR-mediated functional QF2 (QF2+) were calculated. Maternal contribution of Cas9 components in the developing paternal germline cells (far right column) led to 87% of progeny being mutant for GAL4. *GAL4* was almost completely disrupted when male genotypes contained Cas9 components (far left column). Genomic expression of gRNAs *vs.* Cas9 was more potent at increasing the effects of maternal contributions.

*Drosophila* germline stem cells are derived from the pole cells, which are the first cellularized structures in the syncytial embryo (Zalokar and Erk 1976; Foe and Alberts 1983). Pole cells inherit the maternal cytoplasm deposited at the pole plasm (Houston and King 2000). The maternally contributed gRNAs and Cas9 proteins could affect germline stem cells if these components were not excluded from the pole cells. To examine the status of *GAL4* transgenes of F_1_ male germline cells (sperm), we crossed F_1_ males to females containing membrane-targeted GAL4 and QF2 reporters (*UAS-mtdt-3HA* and *QUAS-6xGFP*), and calculated the percentage of progeny containing functional GAL4, *dGAL4*, and functional QF2 (Figure 3 and Figure S1). Strikingly, even in the absence of CRISPR/Cas9 components in the genome, *GAL4* disruption frequency was high (87.2%, *n* = 413 F_2_ flies, Figure 3). This suggests that maternal Cas9 and gRNA are incorporated in the pole cells, and are highly effective at targeting both somatic and germ lines. As expected, maximal frequency of *GAL4* disruption was observed when there was a continuous supply of gRNA and Cas9 protein from the genome (98.2%, *n* = 164 F_2_ males, Figure 3). If gRNAs or Cas9 transgenes are lacking in the genome, the disruption frequencies fall in between (92% and 97%). Interestingly, a continuous supply of gRNA (*vs.* Cas9) appears to be more critical in disrupting *GAL4*, because more intact *GAL4* individuals were observed when *U6*-gRNAs was absent in the F_2_ genome (Figure 3, bar 2 and 3). This could be the result of a shorter half-life of RNA (gRNA) compared to protein (Cas9). Furthermore, Cas9 protein could be supplemented by the translation of preloaded mature Cas9 mRNA transcripts. Finally, consistent with previous observations in somatic cells (Figure 2, B1 and B3), HACK gene conversion events were observed when *QF2^G4H^* donor sequence was present in the genome (Figure 3, bar 1 and 3).

These data demonstrate a form of non-Mendelian inheritance caused by artificial genetic engineering. A maternal effect is defined as the causal influence of the maternal genotype (or phenotype) on the offspring phenotype (Wolf and Wade 2009). In the case reported here, the maternally contributed CRISPR/Cas9 DNA editing components are directly influencing the offspring genotype, which in turn leads to changes in phenotype. Maternal effect mutant phenotypes typically are caused by a lack of maternally contributed RNA or proteins, whereas in this case, effects were caused by the gain of maternally contributed RNA and proteins. As such, we propose calling this form of inheritance a dominant maternal effect.

**Figure 4:**
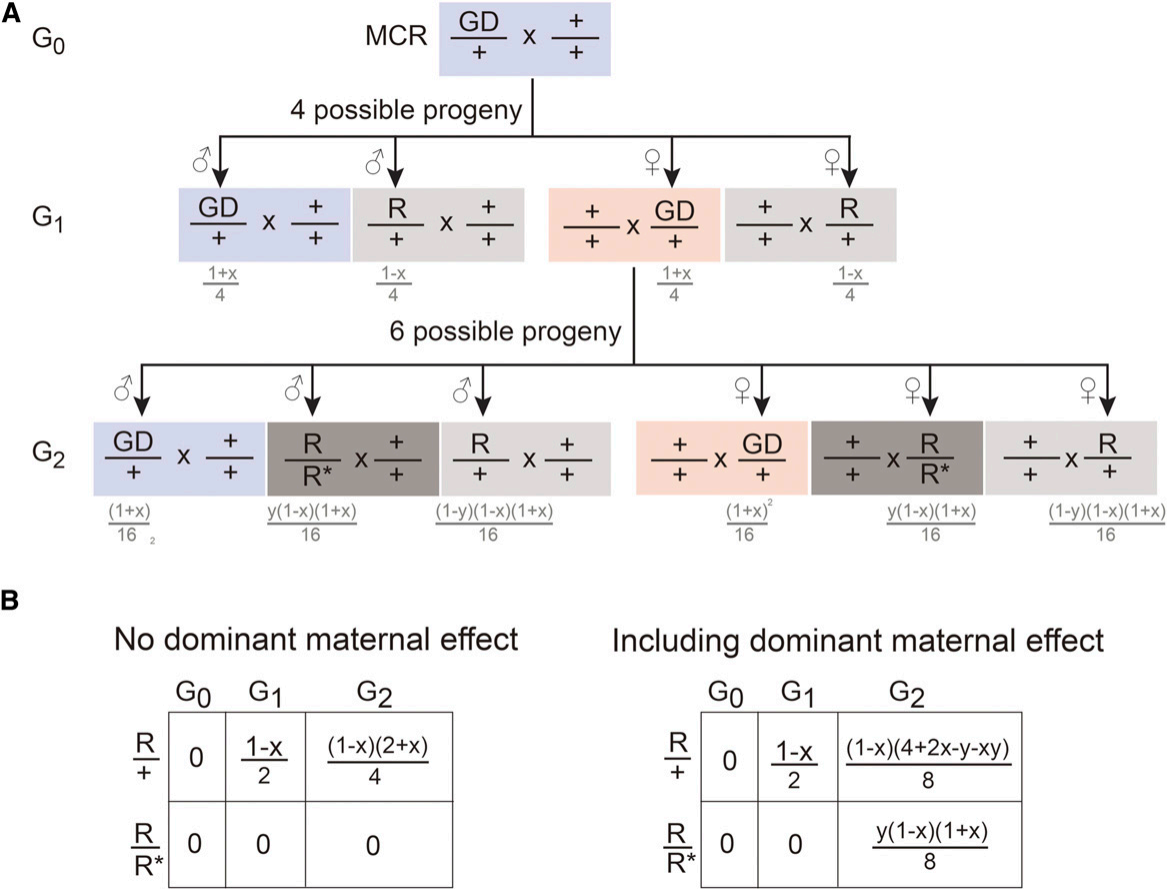
Dominant maternal effects could lead to rapid spreading of resistance with MCR-directed gene drive. (A) Shown are the possible progeny from a transgenic gene drive (GD), containing male animals crossed with wild-type females. If “*x*” indicates the successful rate of GD through MCR, then “‘1 − *x*” is the rate for acquisition of GD resistance by mutation at the target site in the next generation. The consequences of dominant maternal effects are shown in the G_2_ generation, when female individuals contain the MCR-directed GD. If “*y*” indicates the rate of mutation due to dominant maternal effect (leading to GD resistance), then “1 − *y*” is the unaffected rate. Homozygous resistance can occur in a single generation. See Figure S3 for additional details on calculations. (B) Summary calculations comparing GD resistance excluding or including dominant maternal effects when crossed to wild type. For example, if MCR-directed GD efficiency is 95% and maternal dominant effects lead to resistance at 87%, then in the G_2_ generation, heterozygous resistant animals (*R*/+) excluding or including dominant maternal effects is 2.6 or 3.7%, respectively. The frequency of homozygous resistant animals (*R*/R*) excluding or including dominant maternal effects is 0 or 1.06%, respectively. *R* denotes the generation of a resistant mutant through failure of MCR. R* denotes the generation of a resistant mutant obtained by a dominant maternal effect.

MCR-directed gene drive is considered a potential approach for the extermination or control of pest insects, such as the disease transmitting *Aedes aegypti* and *Anopheles gambiae* mosquitoes (Esvelt *et al.* 2014; Gantz *et al.* 2015; Hammond *et al.* 2015). The MCR technique uses the CRISPR/Cas9 system to target and replace a genomic locus with a self-replicating variant containing homology arms flanking Cas9 and gRNA cassettes (Gantz and Bier 2015). By HDR, the variant can copy itself into the wild-type sister chromosome in the genome. It can form the basis of artificial gene drive, whereby a mutant allele is introduced and spread throughout a population. As previously speculated (Port *et al.* 2014; Gantz *et al.* 2015), our results show that CRISPR/Cas9 components will be maternally deposited during oogenesis, and thus could lead to continued CRISPR/Cas9-directed effects even in the absence of genomically supplied DNA editing components. This would not pose a problem if the MCR-directed gene drive success rate were 100%, as the genomic template and CRISPR/Cas9 components would be perfectly coincident. However, since MCR requires HDR in the absence of NHEJ, the actual efficiency ranges from 91 to 99% (Gantz and Bier 2015; Gantz *et al.* 2015; Hammond *et al.* 2015). Our previous work indicated that HDR *vs.* NHEJ efficiency could be as low as ∼10%, depending on the genomic location (*e.g.*, *attP2* locus) (Lin and Potter 2016). This results in mutation of the CRISPR target sites and the generation of heterozygous gene drive–resistant individuals in the progeny (Figure 4, light gray box). Furthermore, the maternal dominant effect highlighted in this work could function to promote the introduction of gene drive–resistant (R*) alleles into the population. This would be most notable when maternally contributed Cas9 and gRNAs are still present but an MCR-compatible Cas9-gRNA cassette is not present in the progeny genome (Figure 4, red box). A homozygous gene drive–resistant individual could be potentially generated from heterozygous gene drive–resistant parents in the natural environment, but the probability would be low if the population density of heterozygous gene drive individuals is also low. The dominant maternal effect described here provides a short-cut that could directly generate homozygous gene drive–resistant individuals when gene drive is not successful in females (Figure 4 and Figure S3). The consequence is potentially rapid generation of homozygous gene drive–resistant animals in the population (Figure 4, dark gray box). Depending on the mechanisms of gene drive to control the population, gene drive–resistant individuals may gain a survival advantage given selective pressure, and counteract MCR-directed gene drive in a population (Gantz *et al.* 2015). These results suggest the most effective gene drive mechanisms will likely depend on supplying CRISPR/Cas9 genomic components only from the father, or simultaneously targeting multiple loci in a gene. Furthermore, models of CRISPR-mediated gene drive (Hammond *et al.* 2015) would need to incorporate dominant maternal effects as an inhibitory mechanism.

## Supplementary Material

Supplemental Material
