# Supplementary material for: Non-Mendelian Dominant Maternal Effects Caused by CRISPR/Cas9 Transgenic Components in Drosophila melanogaster
Source: G3 (Bethesda). 2016 Sep 16;6(11):3685–91. doi: 10.1534/g3.116.034884 (PMC5100867; doi:10.1534/g3.116.034884)
Supplement: Supplemental Material [file supp_6_11_3685__index.html]

Non-Mendelian Dominant Maternal Effects Caused by CRISPR/Cas9 Transgenic Components in Drosophila melanogaster — Supplemental Material 

# Non-Mendelian Dominant Maternal Effects Caused by CRISPR/Cas9 Transgenic Components in *Drosophila melanogaster*

## Supplemental Material for Lin, *et al*, 2016

**Files in this Data Supplement:**

- Figure S1 - Details on genetic crosses used in this study. (.pdf, 120 KB)
- Figure S2 - Cas9 endonuclease is deposited into embryos by a maternal *Act5C-Cas9* transgene. (.pdf, 103 KB)
- Figure S3 - Summaries for estimating dominant maternal effects on gene drive resistance. (.pdf, 162 KB)
