# Supplementary material for: Non-Mendelian Dominant Maternal Effects Caused by CRISPR/Cas9 Transgenic Components in Drosophila melanogaster
Source: G3 (Bethesda). 2016 Sep 16;6(11):3685–91. doi: 10.1534/g3.116.034884 (PMC5100867; doi:10.1534/g3.116.034884)
Supplement: Supplemental Material [file supp_g3.116.034884_FigureS1.pdf]

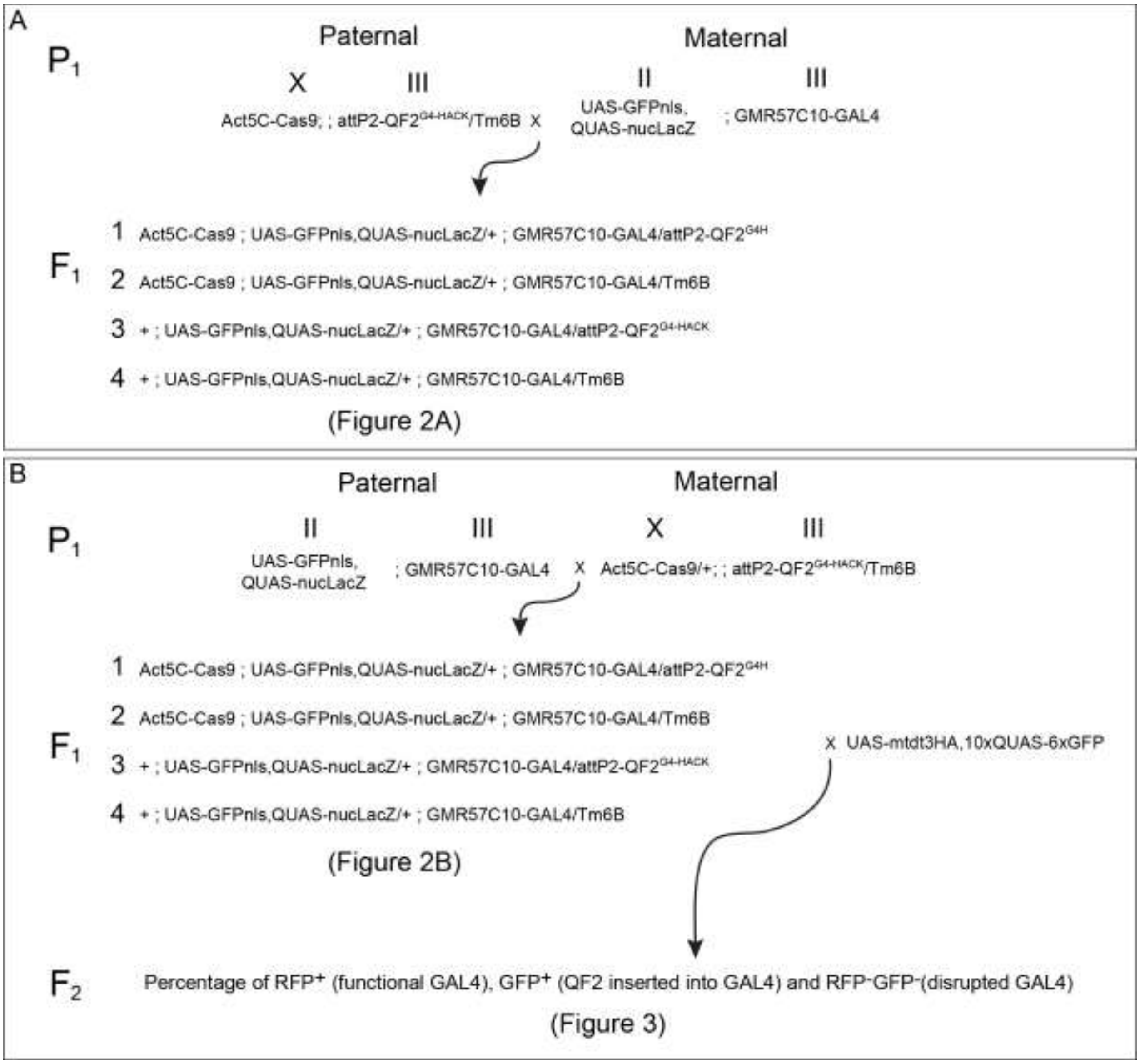

Figure S1. Details on genetic crosses used in this study. Diagramed are the crosses and genotypes for animals used in Figure 2 and Figure 3.
