# Supplementary material for: Non-Mendelian Dominant Maternal Effects Caused by CRISPR/Cas9 Transgenic Components in Drosophila melanogaster
Source: G3 (Bethesda). 2016 Sep 16;6(11):3685–91. doi: 10.1534/g3.116.034884 (PMC5100867; doi:10.1534/g3.116.034884)
Supplement: Supplemental Material [file supp_g3.116.034884_FigureS2.pdf]

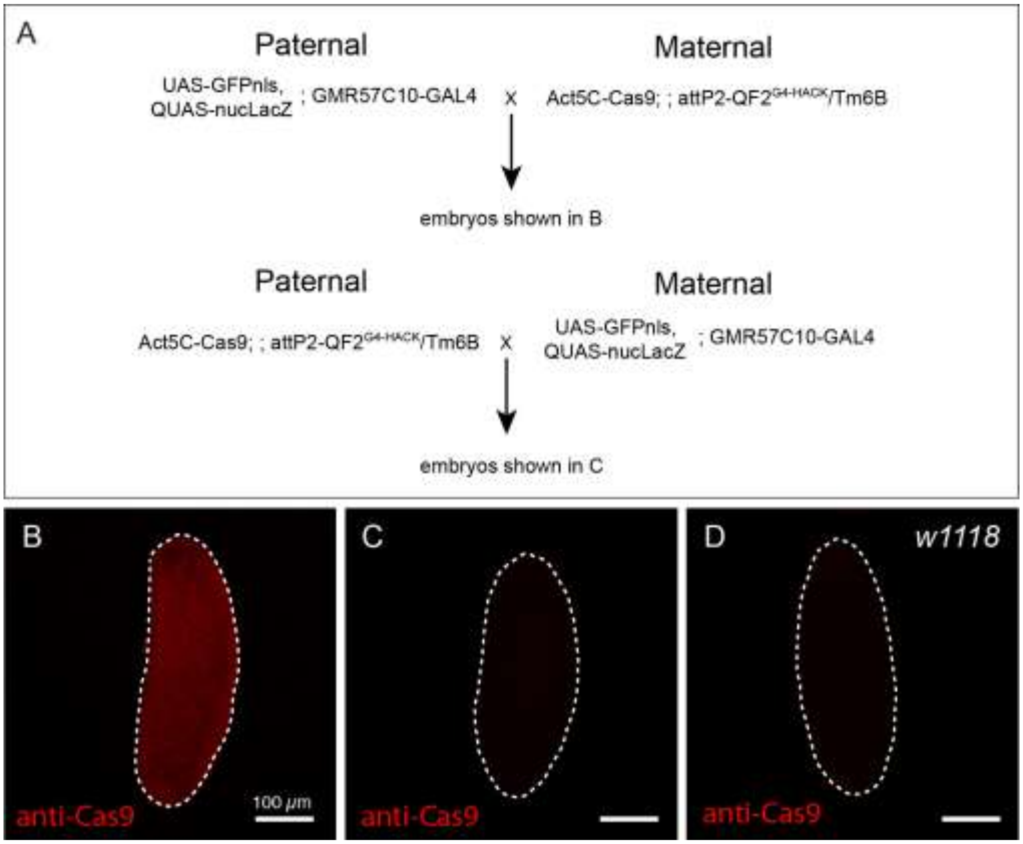

Figure S2. Cas9 endonuclease is deposited into embryos by a maternal *Act5C-Cas9* transgene. A) Schematics of the genetic crosses for the embryos shown in **B** and **C**. **B-D)** Early stage embryo (0-2 hours after egg laying) immunostaining of Cas9 endonuclease. Scale bars, 100 µm.
