# Supplementary material for: Non-Mendelian Dominant Maternal Effects Caused by CRISPR/Cas9 Transgenic Components in Drosophila melanogaster
Source: G3 (Bethesda). 2016 Sep 16;6(11):3685–91. doi: 10.1534/g3.116.034884 (PMC5100867; doi:10.1534/g3.116.034884)
Supplement: Supplemental Material [file supp_g3.116.034884_FigureS3.pdf]

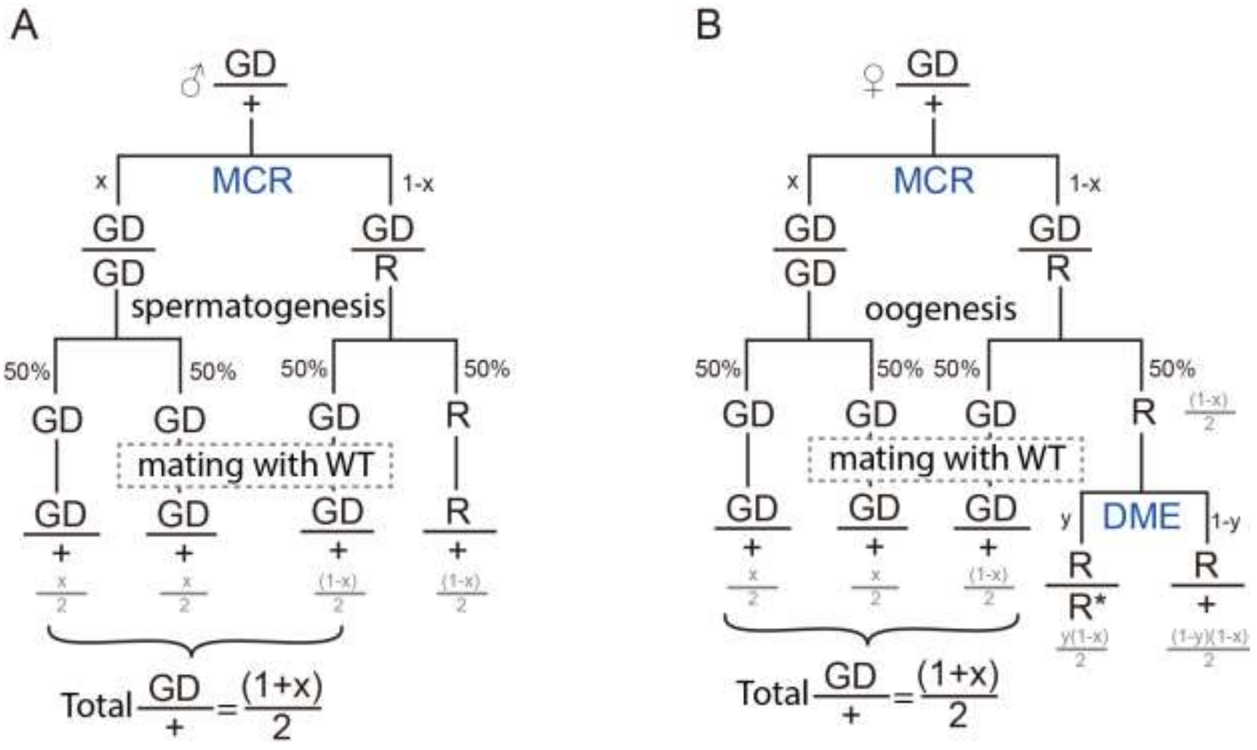

Figure S3. Summaries for estimating dominant maternal effects on gene drive resistance. The effects of MCR-directed gene drive on **A**) male or **B**) female resistant gametes. 'x' indicates the successful rate of Gene Drive through mutagenic chain reaction, and '1-x' is the rate for acquisition of Gene Drive Resistance (R) by mutation at the target site in the next generation. In **B**) female gametes, fertilized eggs containing Resistant gene drive alleles will not have templates for Gene Drive, but will contain maternally deposited Cas9 and gRNAs. This will lead to Cas9/gRNA-directed mutation of the target site at frequency 'y'. Avoiding this dominant maternal effect (DME) can be calculated as '1-y'. R indicates a resistant mutation obtained by failure of MCR. R* indicates a resistant mutation obtained by a dominant maternal effect.
